# Genome-wide association study of medication-use and associated disease in the UK Biobank

**DOI:** 10.1038/s41467-019-09572-5

**Published:** 2019-04-23

**Authors:** Yeda Wu, Enda M. Byrne, Zhili Zheng, Kathryn E. Kemper, Loic Yengo, Andrew J. Mallett, Jian Yang, Peter M. Visscher, Naomi R. Wray

**Affiliations:** 1 Institute for Molecular Bioscience, The University of Queensland, Brisbane, QLD 4072 Australia; 2 Institute for Advanced Research, Wenzhou Medical University, 325027 Wenzhou, Zhejiang China; 3 Department of Renal Medicine, Royal Brisbane and Women’s Hospital, Herston, QLD 4029 Australia; 4 Queensland Brain Institute, The University of Queensland, Brisbane, QLD 4072 Australia

**Keywords:** Genome-wide association studies, Genotype, Genetics research

## Abstract

Genome-wide association studies (GWASs) of medication use may contribute to understanding of disease etiology, could generate new leads relevant for drug discovery and can be used to quantify future risk of medication taking. Here, we conduct GWASs of self-reported medication use from 23 medication categories in approximately 320,000 individuals from the UK Biobank. A total of 505 independent genetic loci that meet stringent criteria (*P* < 10^−8^/23) for statistical significance are identified. We investigate the implications of these GWAS findings in relation to biological mechanism, potential drug target identification and genetic risk stratification of disease. Amongst the medication-associated genes are 16 known therapeutic-effect target genes for medications from 9 categories. Two of the medication classes studied are for disorders that have not previously been subject to large GWAS (hypothyroidism and gastro-oesophageal reflux disease).

## Abstract (web summary)

An understanding of the genetic variants associated with medication use may shed light on the underlying biological pathways of disease, and aid in drug development. Here, Wu and colleagues conduct a GWAS for self-reported medication-use in the UK Biobank, finding more than 500 independent variants and many promising leads for future work.

## Introduction

Susceptibility to most common human diseases is complex and multifactorial, involving genetic, environmental and stochastic factors^1^. During the last decade, large-scale genome-wide association studies (GWASs) have identified thousands of single nucleotide polymorphisms (SNPs) associated with diseases and related traits, consistent with a polygenic genetic architecture of common disease. These results add useful human-relevant information to drug discovery, drug repurposing and clinical trial pipelines^2^. In addition to the valuable disease-diagnoses data, the available medication-use data are also interesting for research. In the context of electronic health record data, medication-use may be an easy route to identify disease-case subjects. However, in clinical practice, it is common that one medication is prescribed for several indications, but conversely, several medications can be prescribed for the same indication. It is likely that medication-use reflects not only similarity between different clinical manifestations^3^ and/or comorbidity^4^ of diseases but also heterogeneity of clinical manifestation (symptoms and signs) and of intervention response (for example, from lifestyle change to the combination of treatments).

We hypothesise that genetic variants associated with taking medications categorised based on anatomical and therapeutic classifications may add additional relevant information to understanding the underlying biological mechanism of diseases and drug development approaches. Here, we study genetic variation in current medication-use using UK Biobank (UKB) (http://www.ukbiobank.ac.uk/about-biobank-uk/) medication data. We report 505 loci independently associated with medication categories. We explore these GWAS findings for biological mechanisms and as drug targets. We estimate the genetic correlation between the 23 medication traits, and with other diseases and traits using published GWAS results. We use Mendelian Randomization (MR) to investigate putative causal relationships among diseases and traits. We show that genetic predisposition to common disease predicts likelihood of taking relevant medications, a significant finding in relation to future practice of precision medicine for common disease. That is, we provide a baseline quantification of an individual’s predicted risk for disease from independent genetic data to their probability of taking disease-relevant medication.

## Results

### UKB medication-taking demographics

There were 502,616 participants (~54% females) with medication records (~73% with non-blank medication information) at their first visit UKB assessment. The mean age for the participants when attending assessment centre was 56.53 (standard deviation (sd) 8.09) years and the mean body mass index (BMI) for participants was 27.43 (sd 4.80). The percentage of participants taking medication increased with age and it was higher for female participants than for males, across all age groups. The percentage of males taking medication increased sharply from ~50% at 40 years old to ~85% at 70 (Supplementary Fig. 1).

### Case-control GWAS of medication-use

**Fig. 1:**
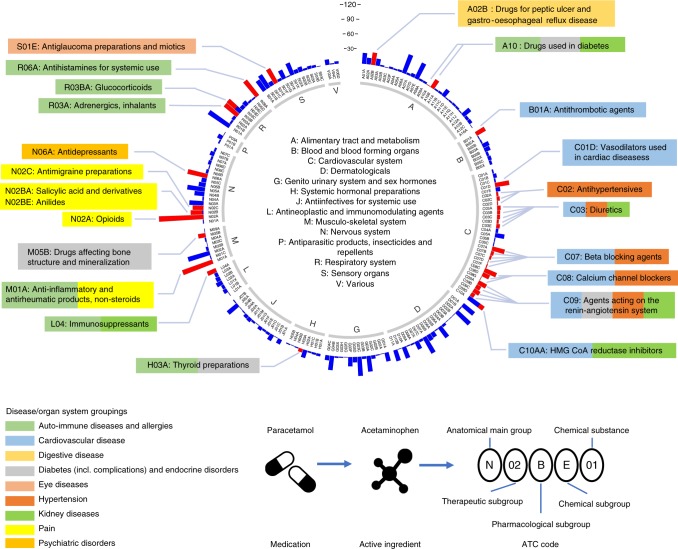
Distribution of 1,752 UKB medications at the first three ATC level. The inner ring corresponds to the first level of the ATC code. The outer ring represents the first three levels of the ATC code (184 subgroups). The length of the bar represents the number of classified UKB medications assigned to that subgroup. Red bars are the 23 medication-taking traits used in analyses (selected based on participant numbers, as shown in Fig. 2). The 23 medication-taking traits are grouped into nine diseases and organ system categories according to the main indications, which is highlighted using different colours (legend at the bottom left). The legend at the bottom right shows how ATC codes are assigned to each UKB medication

**Fig. 2:**
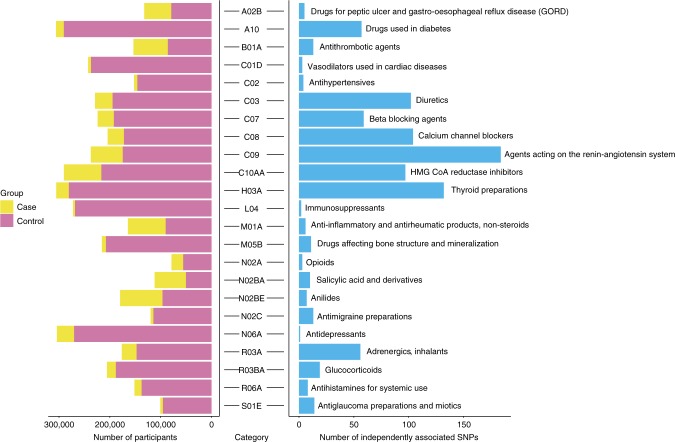
Summary of UKB medication-taking GWAS analyses. For each category, the number of cases and controls is shown on the left and the number of independently-associated SNPs on the right. Text on the right gives the meaning of each medication-taking ATC coded trait

UKB has classified medications into 6,745 categories, of which 1,809 were reported by 10 or more people (Supplementary Fig. 2). Of these 1,752 (97%) were classified using the Anatomical Therapeutic Chemical Classification System^5^ (Fig. 1 and Supplementary Data 1). 318,177 UKB European individuals were selected for 23 medication-use case-control analysis (Supplementary Fig. 3). We conducted a suite of GWASs and post-GWAS analyses (Methods and Supplementary Fig. 4). The medication-use case-control GWASs identify 910 within-trait independent SNPs significantly associated (*P* < 5 × 10^−8^) across 23 medication traits (Fig. 2 and Supplementary Fig. 5). After applying a more stringent multiple testing threshold (*P* < 10^−8^/23)^6^, a total of 505 SNPs remain (Supplementary Table 1 and Supplementary Data 2), with per-trait associations ranging from 0 (C02: hypertensives, N02A:opioids, N06A: antidepressants) to 103 (C09: agents acting on renin-angiotensin system) SNPs. Many of the associated SNPs may simply be a reflection of the primary indication for which the medication is prescribed (Supplementary Table 2). For example, C09 medications have therapeutic effect on hypertension; of the 103 independent SNPs associated with C09 medications (*P* < 10^−8^/23), we identified SNPs previously linked to hypertension (7 SNPs)^7^, systolic blood pressure (32 SNPs)^8^, diastolic blood pressure (5 SNPs)^9^ and pulse pressure (2 SNPs)^9^. Of the 55 independent SNPs associated with C10AA (HMG CoA reductase inhibitors)-associated SNPs (*P* < 10^−8^/23), 19 SNPs have been reported to be significantly associated with low-density lipoprotein cholesterol (LDLC)^10^, supporting the known biological mechanism that statins are effective in lowering LDLC. However, three of the medication classes studied are for disorders that have not previously been subject to large GWAS analysis, including A02B (drugs for peptic ulcer and gastro-oesophageal reflux disease), H03A (thyroid preparations) and N02BE (anilides).

### Genetic risk to common disease predicts medication-taking

**Fig. 3:**
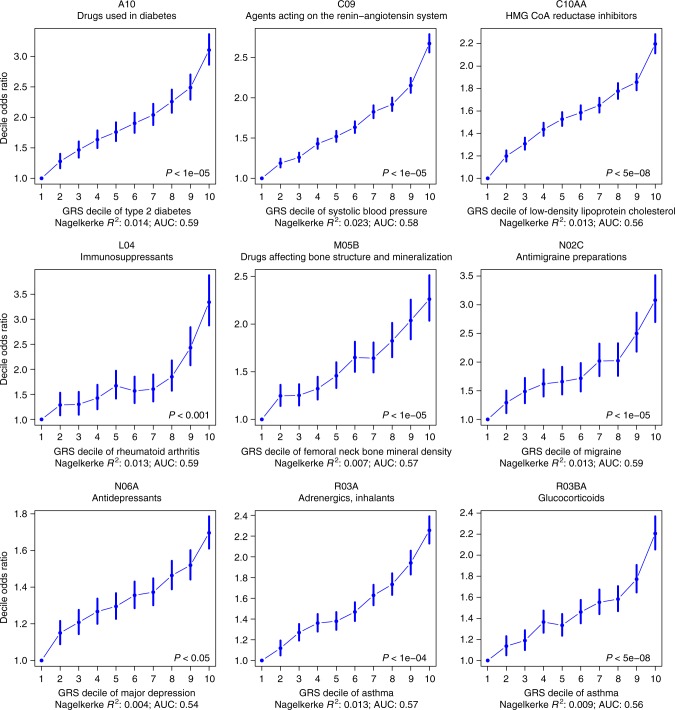
Odds ratio (OR) by genetic risk score (GRS) profile decile. GRS were converted to deciles (1 = lowest, 10 = highest). OR and 95% confidence intervals (blue bars) relative to decile 1 were estimated using logistic regression. The *P* value in the bottom right hand corner of each plot refers to the *P* value threshold in the discovery sample used to generate the GRS. Nagelkerke* R*^2^ represents the proportion of variance of medication-taking explained by the GRS in logistic regression. Note: An increased GRS of femoral neck bone mineral density implies a lower density

We undertook polygenic risk prediction analyses using GWAS summary statistics from eight published disease/traits (Supplementary Table 3) as discovery data to predict disease risk in 9 medication-taking phenotypes as target data. Participants in the UK Biobank with a high genetic risk score (GRS) for different diseases/traits have a higher odds of taking corresponding medications than those with a low GRS (Fig. 3; Supplementary Table 4). The top decile of individuals ranked on risk prediction for depression had an odds ratio (OR) of 1.7 in taking anti-depressants compared to the bottom decile. Similarly comparing top and bottom deciles, we find an OR of 3.1 for taking anti-diabetic medication (A10) for individuals ranked on genetic risk for type 2 diabetes and of 3.3 for taking immunosuppressants (L04) for individuals ranked on their genetic risk for rheumatoid arthritis (RA). The OR increased to 5.2 for taking L04 medications commonly used in RA patients (Supplementary Data 1).

### GWAS results and biological mechanisms

First, we estimated SNP-heritability of the 23 traits using linkage disequilibrium (LD) score regression^11^ (Supplementary Fig. 6; Supplementary Table 5), all traits showed SNP-heritability (proportion of variance attributed to genome-wide SNPs) significantly different from zero to a maximum of 0.15 (s.e. 0.008) for N02A (opioids) on the estimated scale. Second, to identify medication-relevant tissue/cell types, we partitioned the SNP-heritability^12^ based on annotations of SNPs to genes, and genes to differential gene expression between tissues. Among the 23 medication-taking traits, eight traits showed significantly enriched association with genes expressed in at least one tissue at a false discovery rate (FDR) <5% (Supplementary Fig. 7). GWAS associations for thyroid preparations (H03A), immunosuppressants (L04), adrenergics inhalants (R03A), glucocorticoids (R03BA) and antihistamines for systemic use (R06A) were enriched in immune cell types. Those of opioid analgesics (N02A) were enriched in central nervous system tissues, such as limbic system, those of antimigraine preparations (N02C) were enriched in cardiovascular tissue, and those of drugs affecting bone structure and mineralisation (M05B) were enriched in digestive cell type (Supplementary Data 3).

Third, we investigated whether associations between SNPs and medication-taking traits were consistent with mediation through gene expression, based on associations between SNPs and gene expression (eQTLs). We identified 177 unique genes for which expression is significantly associated with 19 medication-taking categories (Supplementary Data 4) using summary data-based Mendelian Randomization (SMR) analysis^13^. Gene-based association tests were conducted using MAGMA^14^ from the GWAS SNP results for each of the 23 medication-taking traits and a total of 1,841 significantly associated unique genes were identified (Supplementary Data 5). To provide biological insights from the GWAS associated loci, we used the gene-based association test summary statistics to test for enrichment in 10,891 gene sets from MSigDB (v5.2)^15,16^. All 23 medication-taking traits were enriched in at least one gene set at FDR <5% (Supplementary Data 6). Several of the results showed plausible relevant biological mechanisms. For example, the genetic associations for taking A10 (drugs used in diabetes) were enriched for the glucose homeostasis gene set, those for taking C10AA (statins) were enriched in the cholesterol homeostasis gene set, C09 (agents acting on renin-angiotensin system) for cardiovascular-related gene sets, M05B (drugs affecting bone structure and mineralisation) for skeletal system development, chondrocyte differentiation gene sets, N02A for gene sets of behavioural response to cocaine and neurogenesis and lastly H03A, L04, R03A, R03BA medications for immune-related gene sets. Interestingly, genes associated with taking A02B (drugs for peptic ulcer and gastro-oesophageal reflux disease) were enriched in gene sets of central nervous system neuron differentiation and of neurogenesis, highlighting the connection between gut and brain^17^.

### Linking medication-taking associated genes to drug targets

Secondary analyses of GWAS results not only provide insights into the biological complexity of common diseases, but also offer opportunities relevant to drug development and repurposing^2,18,19^. To determine whether genes associated with medication-taking could provide clues relevant to drug target identification, we performed analyses using drug-target lists from Santos et al.,^5^ ChEMBL (https://www.ebi.ac.uk/chembl/)^20^ and ClinicalTrials.gov (https://www.clinicaltrials.gov/) database as reference. First, for each UKB medication category, we investigated whether there are therapeutic-effect target genes for medications classified in that medication category; a total of 9 genes were identified (Supplementary Table 6). For example, we find *HMGCR* (Entrez ID: 3156) is, as expected^21^, associated with taking C10AA medications (statins) and encodes the HMGCR protein which is targeted by medications from C10AA category. Second, we tested whether there are therapeutic-effect target genes for treating indications relevant to taking medications of each category; a total of seven genes were identified (Supplementary Table 6). *PCSK9* (Entrez ID: 255738) in our analyses is also associated with taking C10AA medications, and encodes the protein mediating lowering-cholesterol effect of evolocumab (ATC code: C10AX13) and alirocumab (ATC code: C10AX14). Third, we looked at whether there are therapeutic-effect target genes (ever or currently in clinical trial and not approved by U.S. Food and Drug Administration (FDA) yet) for treating indications relevant to medications of each category; a total of eight genes were identified (Supplementary Table 7). For example, *TSLP* (Entrez ID: 85480) is associated with R03A (adrenergics), R03BA (glucocorticoids) and R06A (antihistamines) and also mediates the effect of tezepelumab for the treatment of uncontrolled asthma^22^. Hence, among our associated genes are 24 genes with some known evidence of therapeutic effect. Therefore, we anticipate that other genes that are associated with medication may help to prioritise other putative therapies^23^, but further validation is required. In Supplementary Table 8 we provide additional analyses for two genes, *IDE* and *AGT* that we believe merit further study for type 2 diabetes and C07/C09 related disorders, respectively.

### Genetic correlation between traits and medications

**Fig. 4:**
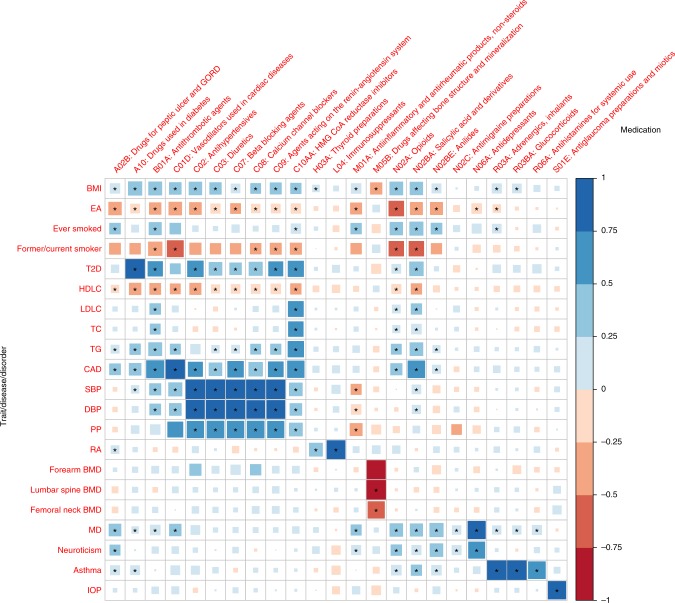
Genetic correlation of the 23 medication-taking traits and 21 diseases/traits. Rows represent traits from Supplementary Table 3 and columns represent 23 medication-taking traits. The size and colour of each square in each cell represent genetic correlation with right-side bar as reference. The significant genetic correlation after correcting for 483 tests (*P* ≤ 1.0 × 10^−4^) are labelled with an asterisk. Abbreviations: Body mass index (BMI), Educational attainment (EA), Type 2 diabetes (T2D), High-density lipoprotein cholesterol (HDLC), Low-density lipoprotein cholesterol (LDLC), Total cholesterol (TC), Triglyceride (TG), Coronary artery disease (CAD), Systolic blood pressure (SBP), Diastolic blood pressure (DBP), Pulse pressure (PP), Rheumatoid arthritis (RA), Bone mineral density (BMD), Major depression (MD), Intraocular pressure (IOP)

The genetic correlation (*r*_*g*_) between the 23 medication-taking traits and 21 traits/diseases (Supplementary Table 3) related to them were calculated using bivariate LD score regression^24^. Many *r*_*g*_ estimated were significantly different from zero. For example, BMI, educational attainment (EA), former/current smoker and coronary artery disease were significantly correlated with most of the medication categories in expected directions. Major depression (MD) and neuroticism showed positive *r*_*g*_ with A02B (gastro-oesophageal reflux drugs), suggesting a link between the brain and the digestive system. Type 2 diabetes showed correlations with taking medications C02, C03, C07~C09 and C10AA, implying a shared genetic architecture of type 2 diabetes, hypertension and hypercholesterolemia. The *r*_*g*_ between B01A and other diseases/traits show similar pattern to those between N02BA medications and other diseases/traits because the original medication aspirin (code number: 1140868226, 59,150 individuals in our analysis) has multiple ATC codes (A01AD05, B01AC06 and N02BA01). Full results are presented in Fig. 4 and Supplementary Data 7.

### Putative causal relationship between traits and medications

**Fig. 5:**
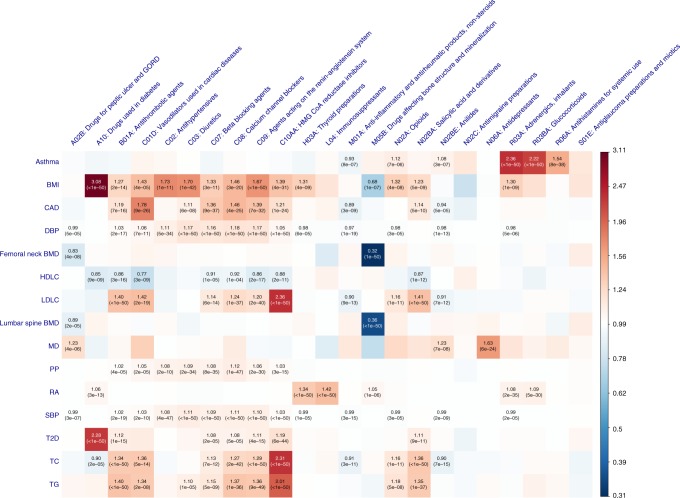
Mendelian Randomization results between 15 diseases/traits and medications. Rows represent 15 diseases/traits as exposure and columns represent 23 medication-taking traits outcome. The significant effects after correcting for 345 tests (*P* ≤ 1.4 × 10^−4^) are labelled with OR (*P* value). The OR is per SD in liability when the exposure is disease. Abbreviation: Body mass index (BMI), Coronary artery disease (CAD), Diastolic blood pressure (DBP), Bone mineral density (BMD), High-density lipoprotein cholesterol (HDLC), Low-density lipoprotein cholesterol (LDLC), Major depression (MD), Pulse pressure (PP), Rheumatoid arthritis (RA), Systolic blood pressure (SBP), Type 2 diabetes (T2D), Total cholesterol (TC), Triglyceride (TG)

It is reasonable to assume that having a disease is causal for taking the associated medication (rather than reverse causation). Therefore, we used MR in a proof-of-principle analysis to quantify causality. Independent SNPs (*P* < 5 × 10^−8^) associated with 15 selected diseases/traits (Supplementary Table 3) were used as instruments to evaluate putative causal relationships^25^ among these 15 diseases/traits and the 23 medication-taking traits (Supplementary Data 8 and Fig. 5). Increasing BMI increases the likelihood of taking A10, B01A, C01D, C02, C03, C07, C08, C09, C10AA, R03A medications, consistent with the role of BMI across diseases related to these medications^25^. The effect of obesity on bone health is controversial^26^. However, results from our analysis clearly show that increasing BMI decreases the likelihood of taking M05B (bone-associated) medications (OR 0.68 per SD of BMI). MD increases the likelihood of taking A02B medication (drugs for peptic ulcer and gastro-oesophageal reflux disease; 1.23-fold increase per SD in liability to MD), capturing a link between the brain and the digestive system. In addition to this, MD increases the likelihood of taking N02BE (1.23-fold increase per SD in liability to MD) medication, which is consistent with comorbidity of pain in some MD patients^27^.

## Discussion

In this study, we profile genetic contributions to medication-use. Traditional GWAS identify DNA variants associated with disease, with a goal that these discoveries ultimately may open the door to new drug treatments. Here, we have taken the reverse approach, aiming to identify DNA variants associated with medication-taking, in recognition that underlying biology may contribute to the same medication being prescribed for several indications, and conversely that only some of those with a given diagnosis may take a particular medication. As expected, some of our results for medication-taking recapitulate GWAS results of the disease traits for which the medication is prescribed. However, we have also identified some associations that may be worthy of follow-up.

We identified 505 linkage disequilibrium independent SNPs associated (*P* *<* 10^−8^/23) with different medication-taking traits. For some of our traits, large GWAS for the medication relevant indications have not been conducted, such as A02B (drugs for peptic ulcer and gastro-oesophageal reflux disease, 2 SNPs) and N02BE (anilides, 4 SNPs). Notably, 76 SNPs were associated with H03A (thyroid preparations—the main indication is hypothyroidism), only 11 of these loci have been previously reported to be associated with hypothyroidism. Conditional (mtCOJO) analysis suggested that these 76 SNPs associated with taking H03A medication are indeed associated with hypothyroidism. We showed that individuals with higher genetic risk of disease have higher likelihood to take relevant medications, for example, individuals with higher GRS for RA have an OR of 3.3 to take immunosuppressants compared with lower GRS individuals (Fig. 3), thereby providing a proof-of-principle validation of precision medicine based upon risk prediction of common diseases, since individuals with high genetic risk of disease can be identified well before the onset of symptoms and the time of medication prescription.

To provide biological insight to the SNP associations for medication-taking^28^, we linked GWAS findings to relevant biological gene sets and drug target efficacy. These analyses generated a series of expected or plausible results, such as genes associated with taking A10 (drugs used in diabetes) enriched in gene sets for glucose homeostasis. Our analyses also generate hypotheses; genes associated with taking N06A (antidepressants) showed enrichment in the gene set for the synthesis and secretion and diacylation of ghrelin, a gut-derived hormone^29^. Previous studies have described an antidepressant-like role of ghrelin^30,31^. This line of evidence suggests that testing a pharmacological effect of ghrelin on depression may be worthwhile. Although medication-associated genes overlapped with only a small proportion of current drug target genes, the framework of genetic association studies provides a potentially valuable resource for new drug target identification and prediction of unfavourable side effects^18^.

Comorbidity is commonly observed in clinical practice, which means the presence of additional diseases in relation to an index disease^32^. Results from genetic correlation and disease-medication (exposure-outcome) MR highlight potential shared etiology, and may help explain medication use in clinical practice. Our analysis showed that major depression increased the likelihood of taking A02B (drugs for peptic ulcer and gastro-oesophageal reflux disease) and N02BE (anilides), the latter consistent with reports that antidepressant prescriptions are not only indicated for depression, but also for pain^33^.

There are a number of limitations in our study. First, although the medication-use data were obtained by trained nurses during interviews, the self-reported nature may limit the accuracy of information. We note that a recent UK study has reported mostly good concordance between prescription data and self-reports of medication taking^34^. Second, the ambiguous names of medications may limit the accurate classification of medications. The reasons (e.g. disease diagnosis) for taking medication were not recorded. The duration, dosage, response and adverse effect of medications were not recorded. For this reason we could not conduct pharmacogenomics analyses to identify associations of SNPs with treatment response or dosage level. Third, our findings are specific to the UK biobank participants, which are recognized to be a non-random sample of the UK population. Fourth, the medication-taking in UK biobank participants may be more representative of medication-taking in the UK and may not translate to other populations and different health systems. Fifth, we would have liked to look for genetic associations with adverse drug reactions, but the number of reported incidents was too few (Supplementary Table 9).

In summary, we identified 505 independent loci associated with different medication-use in 318,177 individuals from UKB, with implications for biological mechanisms, drug target identification and risk of medication use, providing a baseline quantification of the prospect of precision medicine for common disease.

## Methods

### United Kingdom Biobank (UKB)

The study design and sample characteristics of the United Kingdom Biobank (UKB) (http://www.ukbiobank.ac.uk/about-biobank-uk/), a major population-based longitudinal study, have been extensively described elsewhere^35,36^. The UKB was approved by the National Research Ethics Service Committee North West Multi-Centre Haydock and all participants provided written informed consent to participate in the study. Briefly, initial data from more than 500,000 individuals aged 37–73 years were collected between 2006 and 2010, with first-repeat data (revisit) collected on approximately 20,000 individuals from 2012 to 2013 and second-repeat data (imaging visit) collected from ~22,000 individuals since 2014 (Downloaded on March 2017).

### UKB medication classification

Self-reported regular medication and health supplements taken weekly, monthly or three monthly were recorded. Duration and dosage of the medication records were not collected^37^. Medication and health supplements data (Data Field: 20003) were coded using 6,745 categories (Data coding 4). In all, 1,809 of 6,745 categories were for medications taken by at least 10 participants. These 1,809 categories were manually mapped to their corresponding active ingredients using online information (mainly Electronic Medicines Compendium (https://www.medicines.org.uk/emc), Drugs (https://www.drugs.com/), and NetDoctor (https://www.netdoctor.co.uk/)), and were classified using the Anatomical Therapeutic Chemical (ATC) Classification System^5^. Categories named by their active ingredient(s) were directly mapped to the ATC code. Categories named by brand name were first mapped to their active ingredient(s) and then further mapped to ATC code according to the dose and administration route if available. Some categories were ambiguous or could not be mapped to an ATC code, leaving 1,752 categories, which were grouped into 184 subgroups according to the first three ATC levels (Fig. 1 and Supplementary Fig. 2). Supplementary Data 1 provides the active ingredient(s) and ATC code information for the 1,752 categories.

### UKB genotyping, quality control and participants selection

Genotyping details for UKB participants have been reported previously^36^. Briefly, 49,950 participants were genotyped using the UK BiLEVE Axiom Array and 438,427 participants were genotyped using UK Biobank Axiom Array. The Haplotype Reference Consortium (HRC) and UK10K was the imputation reference sample. A European subset (456,414 participants) were identified by projecting the UKB participants onto the 1000 Genome Project principal components coordination. Genotype probabilities were converted to hard-call genotypes using PLINK2 (--hard-call 0.1) and single nucleotide polymorphisms (SNPs) with minor allele count <5, Hardy-Weinberg equilibrium test *P* value < 1 × 10^−6^, missing genotype rate >0.05, or imputation info score <0.3 were excluded. Following the phenotype extraction pipeline for UKB participants provided in Supplementary Fig. 3, 318,177 participants of European ancestry with both genotype and medication records available were selected for further analysis.

### Case-control genome-wide association study (GWAS) designs

Case group and control group were generated according to case medications, similar medications and control medications. Medications with the same ATC levels (at the first two, the first three and the first four levels) were defined as case medications and those taking case medications were assigned to the corresponding case group. Medications of which the first two ATC levels is the same as that of the case medication active ingredients or medications containing case medication active ingredients were defined as similar medications. After excluding participants taking both case medications and similar medications, the remaining participants were assigned to corresponding control group (those taking “99999” category were removed). A total of 23 case-control medication category traits were selected for analysis. Case-control GWAS analyses were conducted using BOLT-LMM^38^ with age, sex, assessment centre and 20 genetic principal components fitted as covariates. 543,919 SNPs generated by linkage disequilibrium (LD) pruning (*r*^2^ < 0.9) from Hapmap3 SNPs were used to control for population structure and polygenic effects. The effect size (*β*) and standard error (se) from BOLT-LMM on the observed 0-1 scale were transformed to odds ratio (OR) and corresponding standard error (SE) using log OR = *β*/(P*(1−*P*)) and SE = se/(*P* *(1−*P*)), where *β* = linear regression coefficient, se = standard error from BOLT-LMM and *P* = case fraction. 7,288,503 SNPs with minor allele frequency (MAF) > 0.01 were analysed. Quasi-independent trait-associated regions were generated through LD clumping retaining the most associated SNP in each region (PLINK (v1.90b)^39,40^ --clump-p1 5e-8 --clump-p2 5e-8 --clump-r2 0.01 --clump-kb 1000). If associated, the MHC region (25Mb-34Mb) was considered as a single locus represented by its most associated SNP. To explore how many SNPs associated with taking medications have been previously linked to their corresponding medication-specific related indications/traits, GCTA (v1.91) was used to perform analyses (--cojo-cond) of 10 medication GWAS summary statistics, conditioning on the given lists of SNPs associated with relevant indications/traits^41,42^. The GWAS Catalog^43^ was used to search published GWASs on relevant indications/traits, with studies selected based on number of independent SNPs reported. To check whether medication-taking associated SNPs were also associated with the main indications for that medication category, GCTA (v1.91) was used to perform analyses (--mtcojo-file) of the 10 medication GWAS summary statistics, conditioning on the related main indications GWAS summary statistics in UKB. The indication phenotype were generated using self-reported non-cancer illness code (Data Field: 20002), main ICD10 diagnoses (Data Field: 41202) and secondary ICD10 diagnoses (Data Field: 41204).

### Genetic risk score (GRS) prediction

Of the 23 medication-taking traits, related published GWAS summary statistics (discovery data) were available for nine of these medication-taking traits (target data), based on eight discovery GWAS studies (Supplementary Table 3). Discovery data were selected as traits related to target data phenotypes, cohort ancestry and with no sample overlap with UKB. The discovery data SNPs were matched with the target data SNPs, then LD pruned and “clumped”, discarding variants within 1,000 kb of, and in *r*^2^ ≥ 0.1 with, another (more significant) marker using SNPs with MAF > 0.01 from 10,000 random sampled unrelated UKB European-ancestry individuals as the LD reference. GRS of target sample individuals were generated for a range of discovery data association *P* value thresholds (5 × 10^−8^, 1 × 10^−5^, 1 × 10^−4^, 0.001, 0.01, 0.05, 0.1, 0.5). For each discovery-target pair, four outcome variables were calculated. (1) The *P* value of case-control GRS difference was calculated by logistic regression. (2) The proportion of variance explained (Nagelkerke* R*^2^) was calculated by comparison of a full model (phenotype~GRS) with a null model (phenotype~1). (3) Area under the receiver operator characteristic curve using R package pROC^44^, which can be interpreted as the probability of ranking a randomly chosen case higher than a randomly chosen control. (4) Odds ratio and 95% confidence interval for the 2nd to 10th GRS deciles group compared with 1st decile. GRS were converted to deciles from lowest (1) to highest (10) GRS.

### LD score regression

Heritability attributable to genome-wide SNPs estimated on the sample scale (SNP-based heritability or $h_{\mathrm{SNP}}^2$) were estimated using LD score regression^11^ from the GWAS summary statistics of 23 medication-taking traits. To evaluate the extent of shared common variant genetic architectures between the 23 medication-taking traits and a range of human traits, disorders and diseases^8,10,45–55^ (Supplementary Table 3), the bivariate genetic correlations^24^ attributable to genome-wide SNPs (*r*_*g*_) were also calculated using LD score regression.

### Linking GWAS findings to gene expression

LD score regression for cell type specific analysis^12^ was applied to test the enrichment heritability in different tissues for each of the 23 medication-taking traits. Gene expression data of 205 tissues (53 from GTEx^56^ and 152 from Franke lab^57,58^) were used for analysis. Summary-data-based Mendelian Randomization (SMR)^13^ was used to identify the causal relationship between gene expression and trait. Westra expression quantitative trait loci (eQTL) data^59^ were used in the SMR analysis.

### Gene-based association and gene sets analysis

MAGMA (v1.06)^14^ was used to compute mean association *P* values for a gene-based test. SNPs with MAF >0.01 from 10,000 random sampled unrelated UKB European-ancestry individuals were used as the LD reference. The window size used was 35 kilobase (kb) upstream and 10 kb downstream to include regulatory elements. The SNPs were mapped to a total of 18,348 genes for each trait using gene locations (build 37) file. For gene sets analysis, curated gene sets (c2.all) and gene ontology sets (c5.bp, c5.cc, c5.mf) from MSigDB (v5.2)^15,16^ were tested for each of the 23 traits. Competitive test *P* values for each gene set were computed; correcting for gene size, density, minor allele count and gene-gene correlations^14^. We generated FDR-adjusted *P* values for biological pathways using Benjamini and Hochberg’s method to account for multiple testing^60^.

### Analyses linking GWAS results to drug target and disease

To check whether associated genes from MAGMA and SMR encode effect-mediating targets for FDA-approved medications or corresponding indications, we used information from Santos et al.,^5^ based on medication approved by the U.S. Food and Drug Administration (FDA) before June 2015. For those approved later, we used the ChEMBL database^20^. To check whether associated genes encode trait-relevant effect-mediating targets for drugs in clinical trial, we used ClinicalTrials.gov (https://www.clinicaltrials.gov/). The CLUE Touchstone tool (https://clue.io/touchstone)^61^ was used to check the correlation between signatures of drugs and knocking down a gene.

### Mendelian Randomization (MR)

MR was used to investigate the causal relationship between the 23 medication-taking traits and other significantly correlated traits. The correlated traits were selected from Supplementary Table 3. We required that the data samples all had ≥7 genome-wide significant loci to use as MR instruments; the median number of SNP instruments was 65. 15 correlated traits were used to conduct MR analysis using Generalized Summary-data-based MR (GSMR)^25^, which includes a heterogeneity test to exclude highly pleiotropic loci. The other parameters were set as default in GCTA-GSMR.

### URLs

UK Biobank: http://www.ukbiobank.ac.uk/about-biobank-uk/; ChEMBL: https://www.ebi.ac.uk/chembl/; ClinicalTrials.gov: https://www.clinicaltrials.gov/; Electronic Medicines Compendium: https://www.medicines.org.uk/emc/; Drugs: https://www.drugs.com/; NetDoctor: https://www.netdoctor.co.uk/; CLUE Touchstone tool: https://clue.io/touchstone.

## Supplementary information

Supplementary Information

Description of Additional Supplementary Files

Supplementary Data 1

Supplementary Data 2

Supplementary Data 3

Supplementary Data 4

Supplementary Data 5

Supplementary Data 6

Supplementary Data 7

Supplementary Data 8

## Data Availability

Summary statistics are available at http://cnsgenomics.com/data.html. The data that support the findings of this study are available from UK Biobank (http://www.ukbiobank.ac.uk/about-biobank-uk/). Restrictions apply to the availability of these data, which were used under license for the current study (Project ID: 12514). Data are available for bona fide researchers upon application to the UK Biobank.
