# Supplementary material for: Genome-wide association study of medication-use and associated disease in the UK Biobank
Source: Nat Commun. 2019 Apr 23;10:1891. doi: 10.1038/s41467-019-09572-5 (PMC6478889; doi:10.1038/s41467-019-09572-5)
Supplement: Supplementary file 1 — Supplementary Information [file 41467_2019_9572_MOESM1_ESM.pdf]

Supplementary Information for

Genome-wide association study of medication-use and associated disease in the UK Biobank

Wu *et al.*

Supplementary Figures

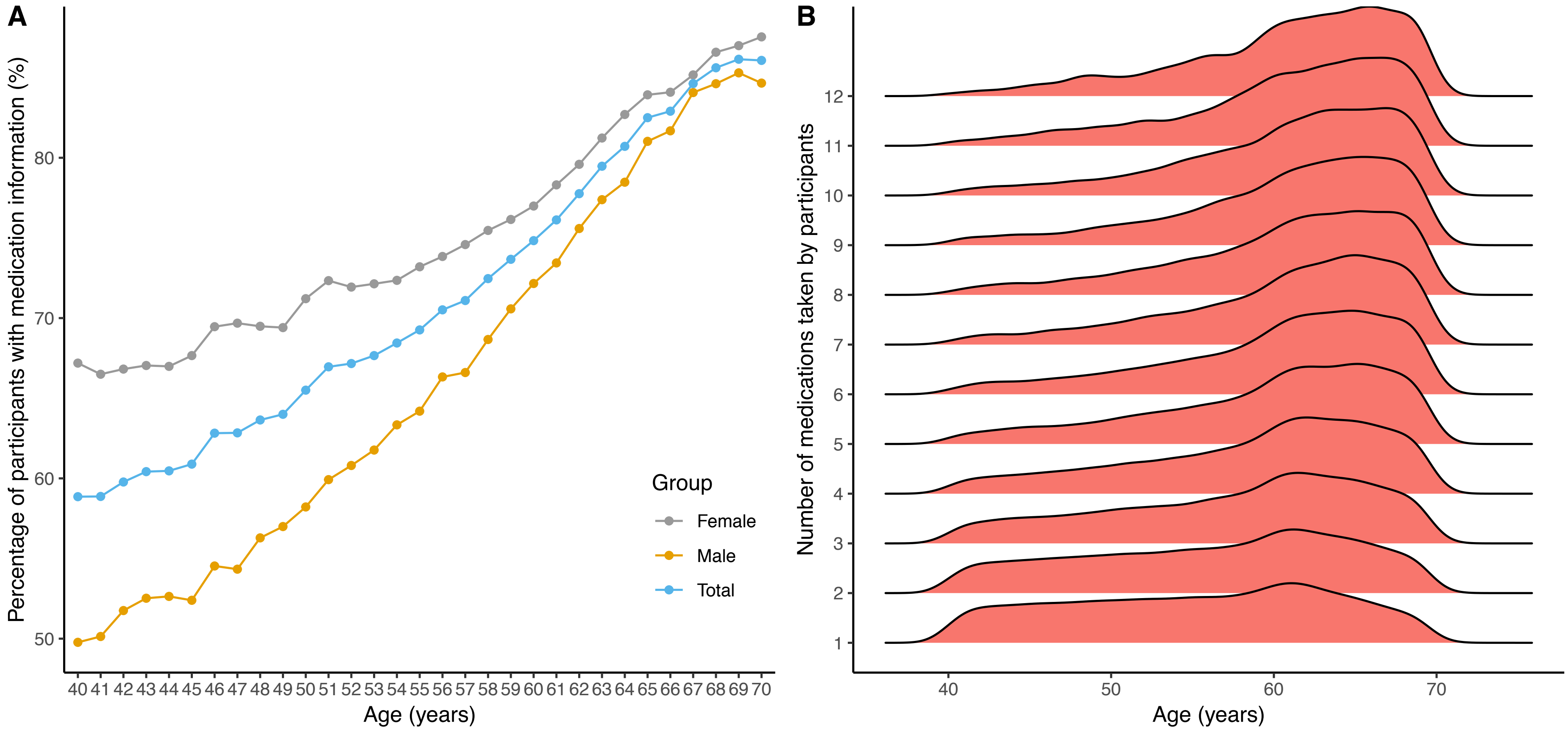

Supplementary Figure 1. Demographic statistics for participants who completed self-report medication questionnaires at first visit in Biobank. A. Percentage of participants with medication information at different ages. B. Age distribution of participants stratified by the number of medications taken.

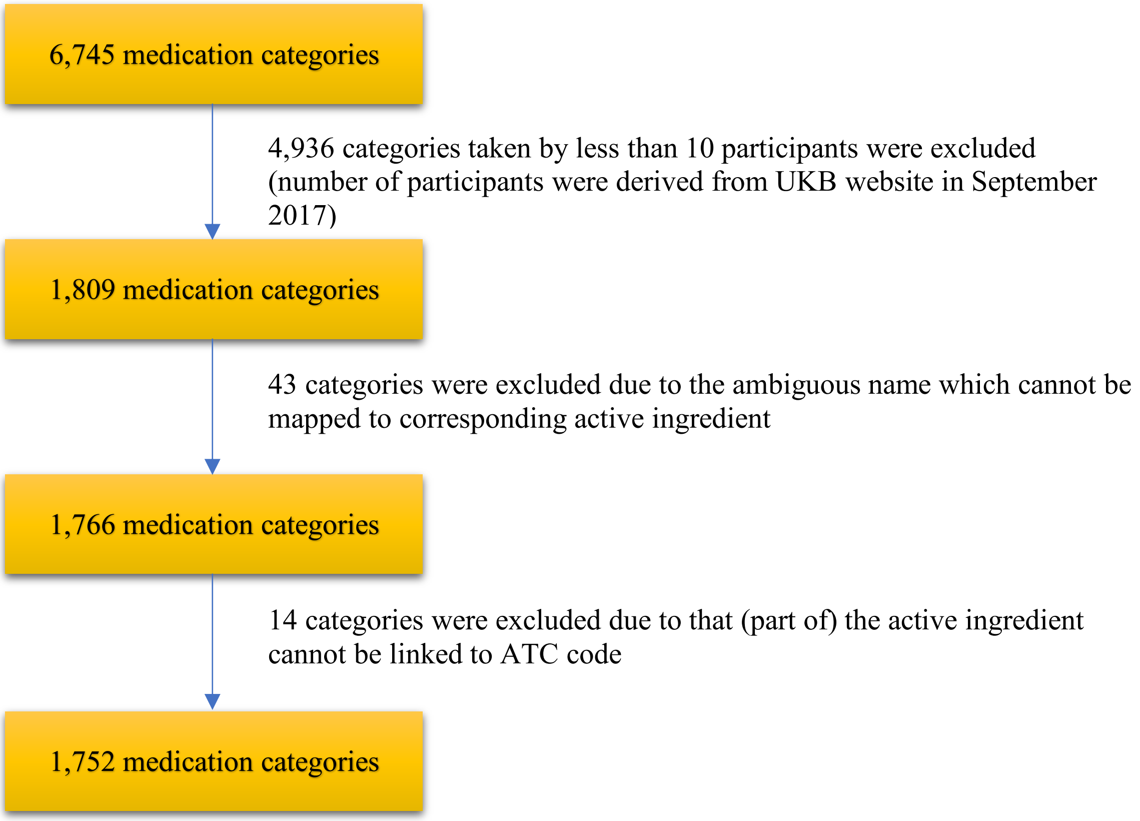

Supplementary Figure 2. Analysis pipeline of UKB medication.

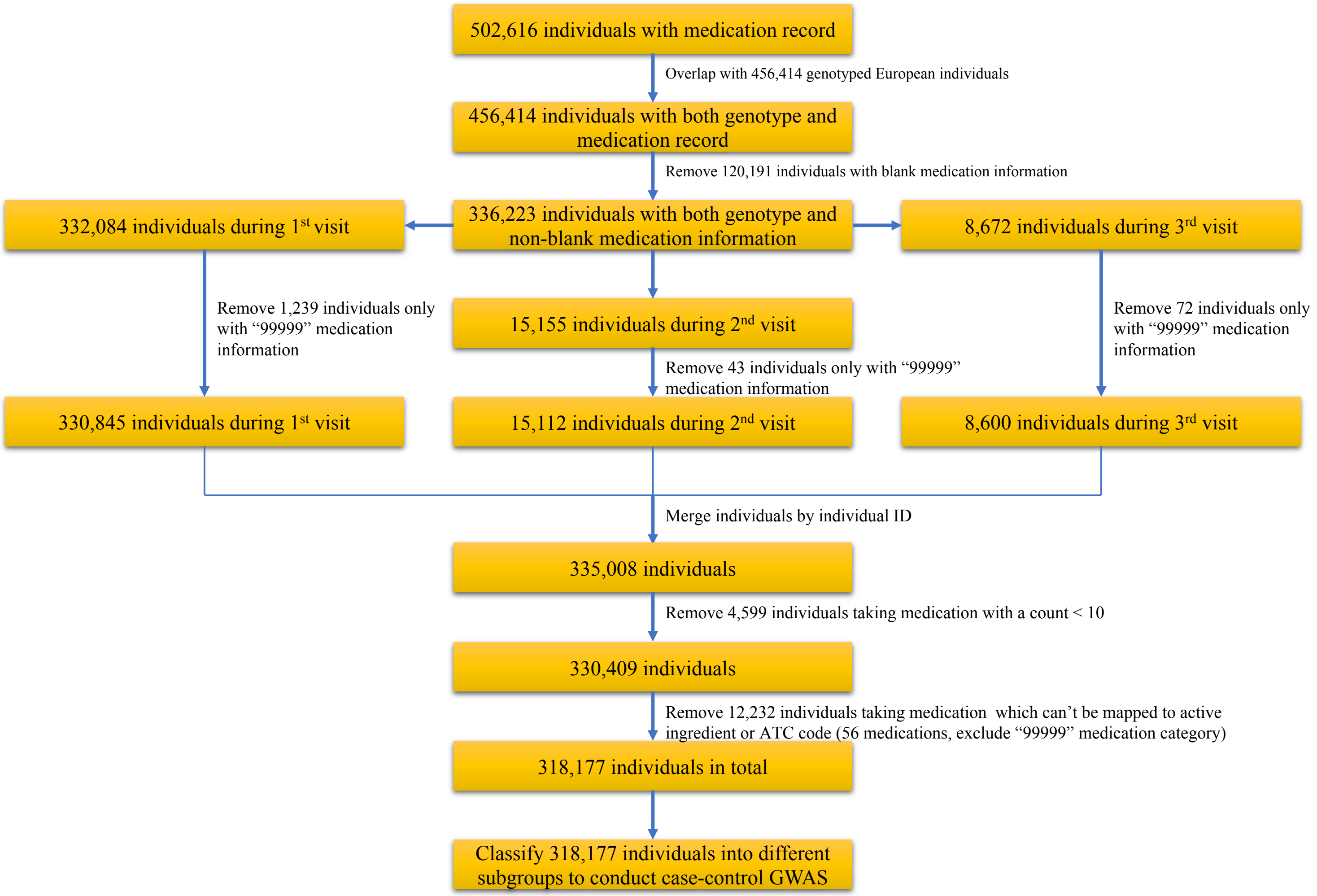

Supplementary Figure 3. Phenotype extraction pipeline for UKB participants.

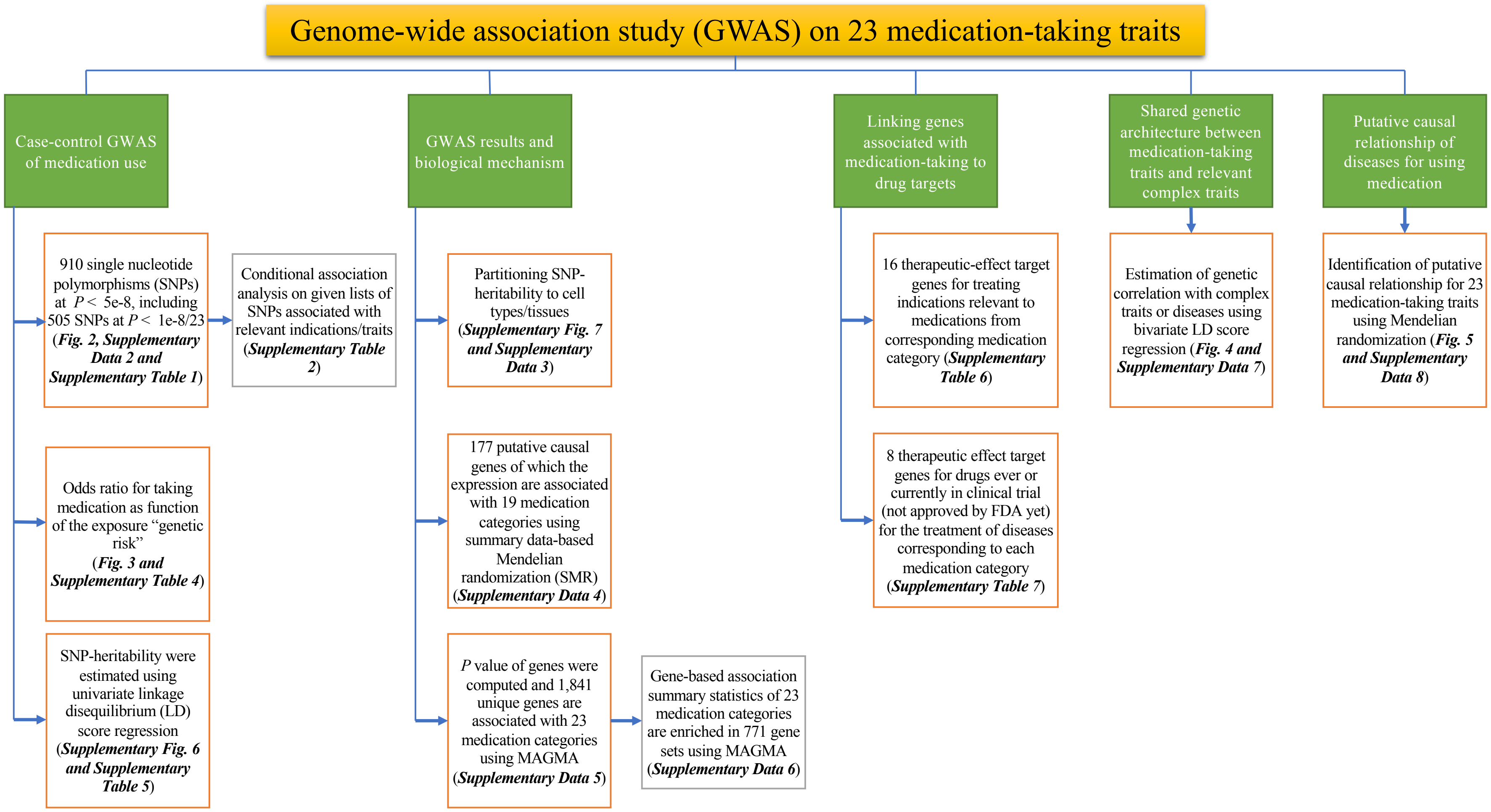

Supplementary Figure 4. Full analysis workflow of the study.

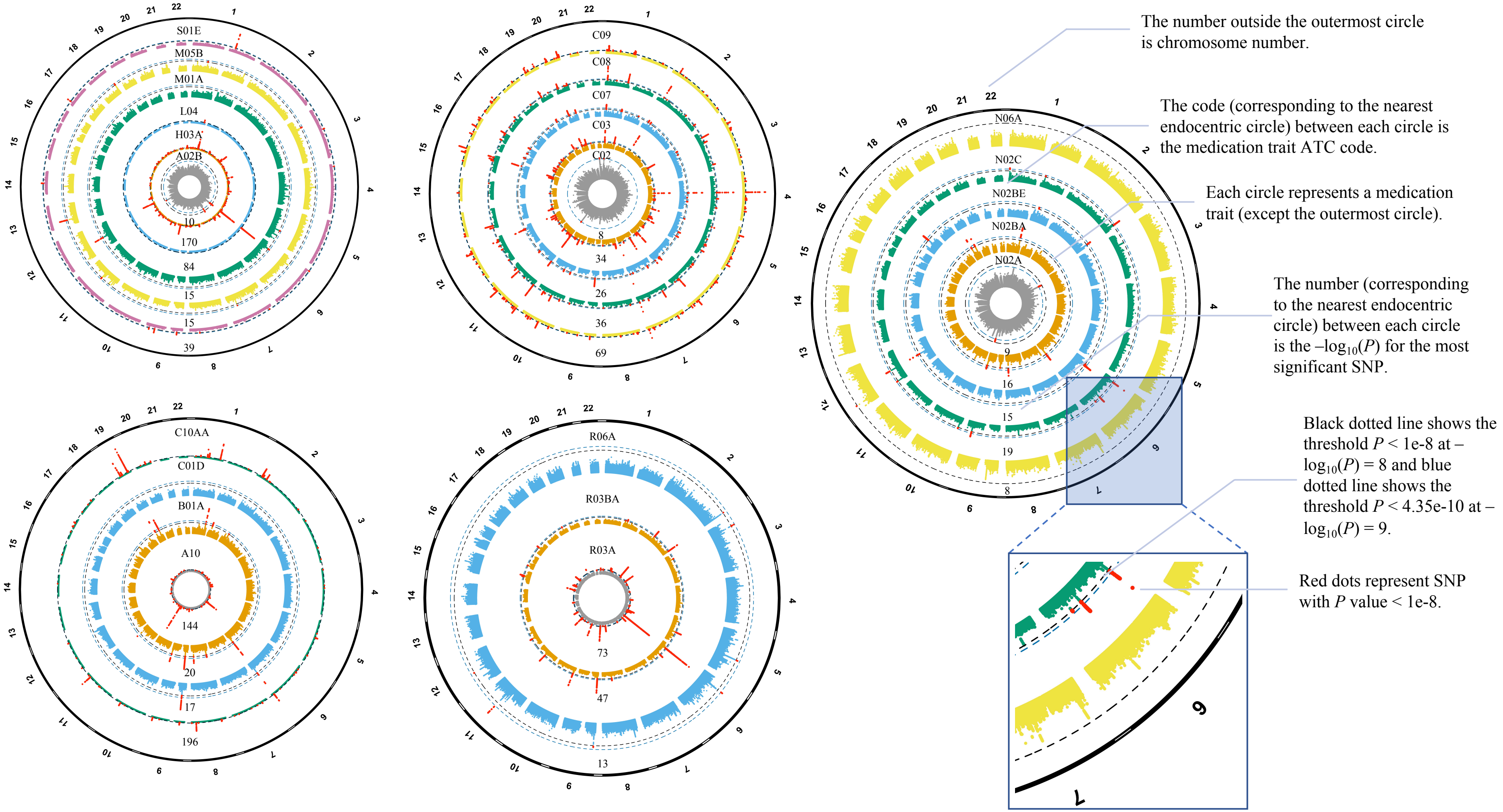

Supplementary Figure 5. Manhattan plot for 23 medication-taking traits.

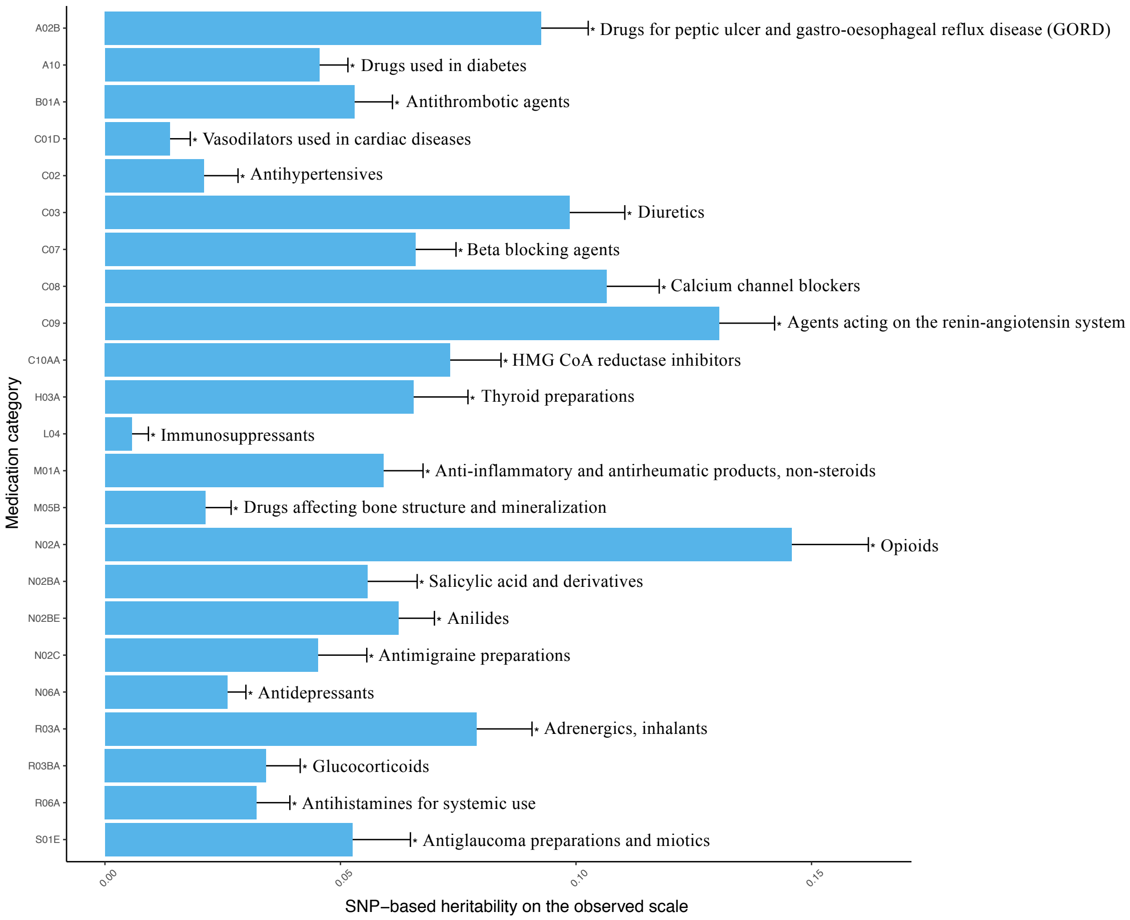

Supplementary Figure 6. The SNP-based heritability of medication-taking trait. Texts on the right side represent each medication-taking trait. Error bar represent the upper 95% confidence interval for the observed SNP-based heritability of each medication-taking trait. The significant level were labelled as an asterisk after 23 tests ($P < 0.05/23$). The SNP-based heritability were estimated from linkage disequilibrium (LD) score regression[1].

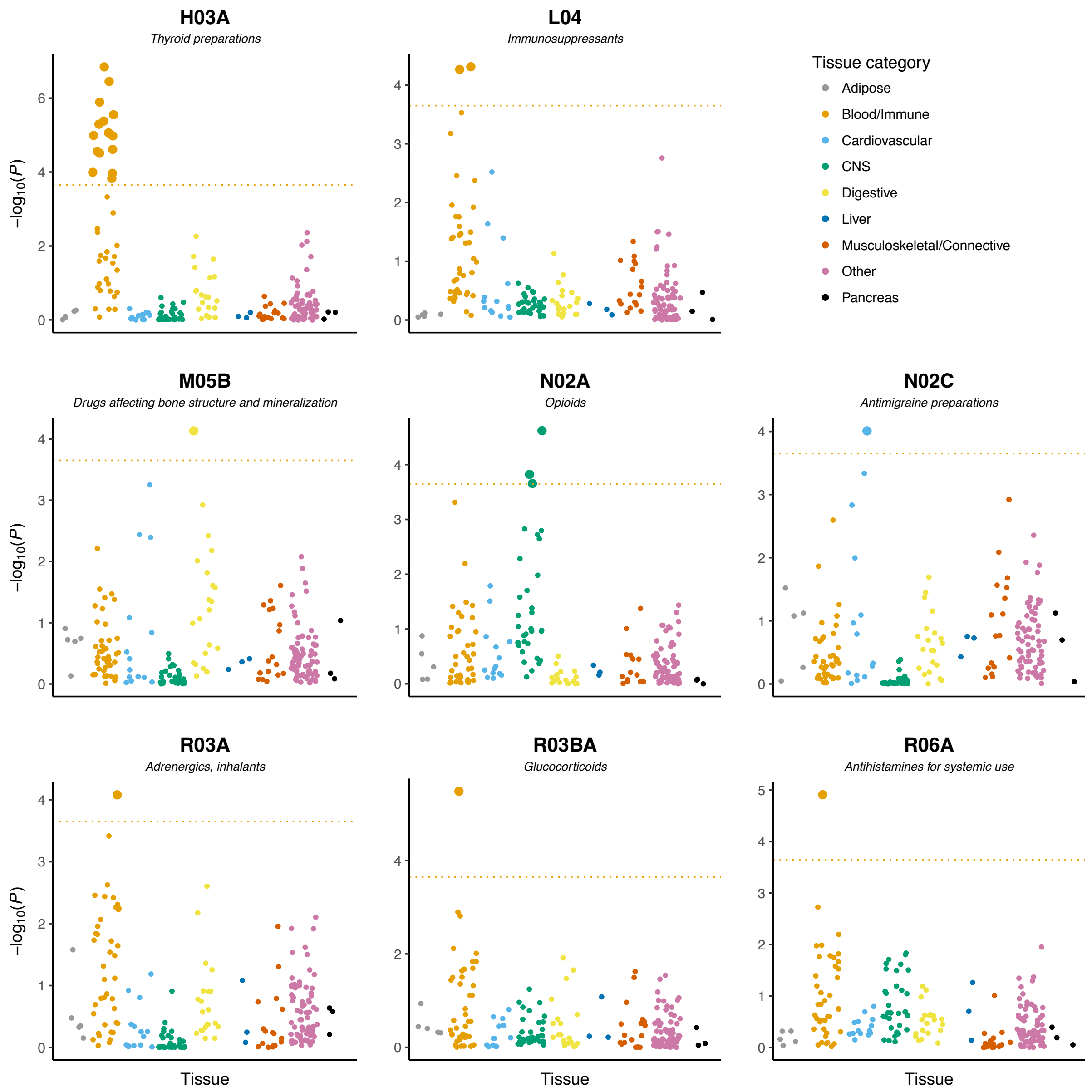

Supplementary Figure 7. Results of the multiple-tissue analysis for 8 medication-taking traits. Each dot represents a tissue or cell type. The dotted line shows the threshold of FDR < 5% corresponding to -$\log_{10}(P)$ = 3.65. Large dots pass the threshold.

Supplementary Tables

Supplementary Table 1. Summary of medication GWAS.

#	Medication category	Name	No. of case	No. of control	No. of total	No. independent GWAS hit ($P \leq$ 5E-8)	No. independent GWAS hit ($P <$ 1E-8/23)
1	A02B	Drugs for peptic ulcer and gastro-oesophageal reflux disease (GORD)	53137	79230	132367	5	2
2	A10	Drugs used in diabetes	15272	290641	305913	57	33
3	B01A	Antithrombotic agents	67653	85986	153639	13	11
4	C01D	Vasodilators used in cardiac diseases	5546	237113	242659	3	3
5	C02	Antihypertensives	6431	145949	152380	4	0
6	C03	Diuretics	34453	194633	229086	102	60
7	C07	Beta blocking agents	31700	192324	224024	59	31
8	C08	Calcium channel blockers	31904	172474	204378	104	51
9	C09	Agents acting on the renin-angiotensin system	62752	174778	237530	184	103
10	C10AA	HMG CoA reductase inhibitors	73475	216910	290385	97	55
11	H03A	Thyroid preparations	24832	280750	305582	132	76
12	L04	Immunosuppressants	3954	268648	272602	2	2
13	M01A	Anti-inflammatory and antirheumatic products, non-steroids	74150	90370	164520	6	1
14	M05B	Drugs affecting bone structure and mineralization	7870	207798	215668	11	1
15	N02A	Opioids	22982	55826	78808	3	0
16	N02BA	Salicylic acid and derivatives	61583	50427	112010	10	7
17	N02BE	Anilides	83218	96592	179810	7	4
18	N02C	Antimigraine preparations	5521	114323	119844	13	6
19	N06A	Antidepressants	33757	270405	304162	1	0
20	R03A	Adrenergics, inhalants	28880	147565	176445	56	35
21	R03BA	Glucocorticoids	17352	188348	205700	19	15
22	R06A	Antihistamines for systemic use	13984	137652	151636	8	2
23	S01E	Antiglaucoma preparations and miotics	5215	95653	100868	14	7

Supplementary Table 2. Number of medication-associated independent loci that have been linked to diseases relevant to the medication.

#	Medication category	No. of independent SNPs (*P* < 1E-8/23) for that medication category	External						UKB		
			Related indication/trait	PMID	Initial sample size [a]	Replication sample size [a]	No. of independent SNPs reported [a]	Number of medication-associated independent loci that have been previously linked to diseases relevant to the medication [b]	Related indication/trait	Number of medication-associated independent loci that are also associated with related disease	SNPs remain significant after mtCOJO analyses
1	A10: Drugs used in Diabetes	33	Type 1 diabetes	19430480	7,514 European ancestry cases, 9,045 European ancestry controls	4,267 European ancestry cases, 4,670 European ancestry controls, 4,342 European ancestry trios from 2,319 families	38	1	Type 2 diabetes	32	rs9273364
			Type 2 diabetes	28869590	6,353 South Asian ancestry cases, 7,179 South Asian ancestry controls, 3,871 European ancestry cases, 16,427 European ancestry controls, 34,840 cases, 114,981 controls	7,888 South Asian ancestry cases, 20,679 South Asian ancestry controls, 387 European ancestry cases, 2,092 European ancestry controls, 19,998 East Asian ancestry cases, 30,983 East Asian ancestry controls	157	23			
2	C03: Diuretics	60	Hypertension	21909115	69,395 European ancestry individuals	Up to 133,361 European ancestry individuals	11	7	Hypertension	59	rs916880
			Systolic blood pressure	28739976	150,134 European ancestry individuals	87,359 European ancestry individuals, 140,886 European and unknown ancestry individuals	96	26			
			Diastolic blood pressure	28135244	140,886 European ancestry individuals	190,318 European ancestry individuals	94	2			
			Pulse pressure	28135244	140,886 European ancestry individuals	190,318 European ancestry individuals	94	0			
3	C07: Beta blocking agents	31	Hypertension	21909115	69,395 European ancestry individuals	Up to 133,361 European ancestry individuals	11	5	Hypertension	30	rs2891168
			Systolic blood pressure	28739976	150,134 European ancestry individuals	87,359 European ancestry individuals, 140,886 European and unknown ancestry individuals	96	14			
			Diastolic blood pressure	28135244	140,886 European ancestry individuals	190,318 European ancestry individuals	94	2			
			Pulse pressure	28135244	140,886 European ancestry individuals	190,318 European ancestry individuals	94	1			
4	C08: Calcium channel blockers	51	Hypertension	21909115	69,395 European ancestry individuals	Up to 133,361 European ancestry individuals	11	4	Hypertension	50	rs7983337
			Systolic blood pressure	28739976	150,134 European ancestry individuals	87,359 European ancestry individuals, 140,886 European and unknown ancestry individuals	96	20			
			Diastolic blood pressure	28135244	140,886 European ancestry individuals	190,318 European ancestry individuals	94	2			
			Pulse pressure	28135244	140,886 European ancestry individuals	190,318 European ancestry individuals	94	0			

5	C09: Agents acting on the renin-angiotensin system	103	Hypertension	21909115	69,395 European ancestry individuals	Up to 133,361 European ancestry individuals	11	7	Hypertension	100	rs4970834 | rs10217586 | rs7412
			Systolic blood pressure	28739976	150,134 European ancestry individuals	87,359 European ancestry individuals, 140,886 European and unknown ancestry individuals	96	32			
			Diastolic blood pressure	28135244	140,886 European ancestry individuals	190,318 European ancestry individuals	94	5			
			Pulse pressure	28135244	140,886 European ancestry individuals	190,318 European ancestry individuals	94	2			
6	C10AA: HMG CoA reductase inhibitors	55	LDL cholesterol	24097068	94,595 European ancestry individuals	93,982 European ancestry individuals	58	19	Hypercholesterolemia	53	rs7903146 | rs1537371
			HDL cholesterol	24097068	94,595 European ancestry individuals	93,982 European ancestry individuals	71	9			
			Cholesterol, total	24097068	94,595 European ancestry individuals	93,982 European ancestry individuals	73	18			
			Triglycerides	24097068	94,595 European ancestry individuals	93,982 European ancestry individuals	41	8			
7	H03A: Thyroid preparations	76	Hypothyroidism	27182965	17,558 European ancestry cases, 117,083 European ancestry controls	-	26	11	Hypothyroidism	76	-
8	R03A: Adrenergics, inhalants	35	Asthma	29273806	19,954 European ancestry cases, 107,715 European ancestry controls, 2,149 African ancestry cases, 6,055 African ancestry controls, 1,239 Japanese ancestry cases, 3,976 Japanese ancestry controls, 606 Latino cases, 792 Latino controls	-	51	13	Asthma	35	-
9	R03BA: Glucocorticoids	15	Asthma	29273806	19,954 European ancestry cases, 107,715 European ancestry controls, 2,149 African ancestry cases, 6,055 African ancestry controls, 1,239 Japanese ancestry cases, 3,976 Japanese ancestry controls, 606 Latino cases, 792 Latino controls	-	51	8	Asthma	15	-
10	S01E: Antiglaucoma preparations and miotics	7	Glaucoma (primary open-angle)	25173105	1,155 European ancestry cases, 1,922 European ancestry controls	3,548 European ancestry cases, 9,496 European ancestry controls	9	3	Glaucoma	7	-
			Intraocular pressure	28073927	25,916 European ancestry individuals, 1,073 Orcadian (founder/genetic isolate) individuals, 2,589 Erasmus Rucphen (founder/genetic isolate) individuals	8,352 Asian ancestry individuals	9	2			

[a] Information are derived from GWAS Catalog (https://www.ebi.ac.uk/gwas/).
[b] Analyses were performed using GCTA --cojo-cond function, together with medication-taking GWAS summary statistics and relevant indication/trait associated independent SNPs as input. Number of independent medication-associated SNPs which are not significant after the analyses are counted.

Supplementary Table 3. Downloaded GWAS summary statistics for further analysis.

Trait	Title	Title URL	Published year	PMID	Genetic correlation analysis	Genetic risk score analysis	Mendelian Randomization analysis	Note
Body mass index (BMI)	Genetic studies of body mass index yield new insights for obesity biology	https://www.nature.com/articles/nature14177	2015	25673413	✓		✓	
Educational attainment (EA)	Genome-wide association study identifies 74 loci associated with educational attainment	https://www.nature.com/articles/nature17671	2016	27225129	✓			
Ever vs never smoked	Genome-wide meta-analyses identify multiple loci associated with smoking behavior	https://www.nature.com/articles/ng.571	2010	20418890	✓			
Former vs current smoker					✓			
Type 2 diabetes (T2D)	Large-scale association analysis provides insights into the genetic architecture and pathophysiology of type 2 diabetes	https://www.nature.com/articles/ng.2383	2012	22885922	✓	✓	✓	
High-density lipoprotein cholesterol (HDLC)	Discovery and refinement of loci associated with lipid levels	https://www.nature.com/articles/ng.2797	2013	24097068	✓		✓	
Low-density lipoprotein cholesterol (LDLC)					✓	✓	✓	
Total cholesterol (TC)					✓		✓	
Triglycerides (TG)					✓		✓	
Coronary artery disease (CAD)	Large-scale association analysis identifies 13 new susceptibility loci for coronary artery disease	https://www.nature.com/articles/ng.784	2011	21378990	✓		✓	
Systolic blood pressure (SBP)	Novel blood pressure locus and gene discovery using genome-wide association study and expression data sets from blood and the kidney	http://hyper.ahajournals.org/content/hypertensionaha/70/3/e4.full.pdf	2017	28739976	✓	✓	✓	
Diastolic blood pressure (DBP)					✓		✓	
Pulse pressure (PP)					✓		✓	
Rheumatoid arthritis (RA)	Genetics of rheumatoid arthritis contributes to biology and drug discovery	https://www.nature.com/articles/nature12873	2014	24390342	✓	✓	✓	
Forearm bone mineral density	Whole-genome sequencing identifies EN1 as a determinant of bone density and fracture	https://www.nature.com/articles/nature14878	2015	26367794	✓			
Femoral neck bone mineral density					✓	✓	✓	
Lumbar spine bone mineral density					✓		✓	
Migraine	Meta-analysis of 375,000 individuals identifies 38 susceptibility loci for migraine	https://www.nature.com/articles/ng.3598	2016	27322543		✓		
Major depression (MD)	Genome-wide association analyses identify 44 risk variants and refine the genetic architecture of major depression	https://www.nature.com/articles/s41588-018-0090-3	2018	29700475	✓	✓	✓	Excluding the UKB cohort
Neuroticism	Genetic variants associated with subjective well-being, depressive symptoms, and neuroticism identified through genome-wide analyses	https://www.nature.com/articles/ng.3552	2016	27089181	✓			
Asthma	Multi-ancestry association study identifies new asthma risk loci that colocalize with immune-cell enhancer marks	https://www.nature.com/articles/s41588-017-0014-7	2018	29273806	✓	✓	✓	
Intraocular pressure (IOP)	New insights into the genetics of primary open-angle glaucoma based on meta-analyses of intraocular pressure and optic disc characteristics	https://academic.oup.com/hmg/article/26/2/438/2970289	2017	28073927	✓			

Supplementary Table 4. Results of genetic risk score of 8 diseases/traits for 9 medication-taking phenotypes.

Discovery trait [a]	Target trait	Prediction *P* value range	Target sample size	Proportion of cases	*Nagelkerke* R^2	*P* value	AUC	OR_{10} [b]	OR_{10} CI [c]
Type 2 diabetes (T2D)	A10: Drugs used in diabetes	0~5.00E-08	305913	0.050	0.012	8.0E-267	0.581	2.78	2.57~3.00
		0~1.00E-05			0.014	4.7E-311	0.588	3.10	2.86~3.36
		0~1.00E-04			0.012	6.1E-265	0.581	2.75	2.55~2.98
		0~1.00E-03			0.009	2.5E-199	0.570	2.40	2.23~2.59
		0~1.00E-02			0.007	2.8E-159	0.563	2.19	2.03~2.37
		0~5.00E-02			0.007	2.1E-158	0.563	2.23	2.07~2.41
		0~1.00E-01			0.007	7.3E-144	0.560	2.24	2.07~2.42
		0~5.00E-01			0.006	5.8E-140	0.559	2.25	2.08~2.43
Systolic blood pressure (SBP)	C09: Agents acting on the renin-angiotensin system	0~5.00E-08	237530	0.264	0.017	0.0E+00	0.568	2.39	2.29~2.49
		0~1.00E-05			0.023	0.0E+00	0.580	2.67	2.56~2.79
		0~1.00E-04			0.023	0.0E+00	0.580	2.70	2.59~2.82
		0~1.00E-03			0.023	0.0E+00	0.580	2.80	2.68~2.92
		0~1.00E-02			0.021	0.0E+00	0.578	2.67	2.56~2.78
		0~5.00E-02			0.019	0.0E+00	0.577	2.60	2.49~2.71
		0~1.00E-01			0.018	0.0E+00	0.576	2.51	2.41~2.62
		0~5.00E-01			0.016	0.0E+00	0.573	2.39	2.29~2.49
Low-density lipoprotein cholesterol (LDLC)	C10AA: HMG CoA reductase inhibitors	0~5.00E-08	290385	0.253	0.013	0.0E+00	0.560	2.20	2.11~2.28
		0~1.00E-05			0.011	0.0E+00	0.557	2.08	2.00~2.17
		0~1.00E-04			0.010	0.0E+00	0.553	1.91	1.84~1.98
		0~1.00E-03			0.006	4.2E-262	0.541	1.69	1.63~1.76
		0~1.00E-02			0.003	2.9E-129	0.529	1.42	1.37~1.48
		0~5.00E-02			0.002	4.5E-89	0.524	1.34	1.29~1.39
		0~1.00E-01			0.002	4.1E-77	0.523	1.32	1.27~1.37
		0~5.00E-01			0.001	2.4E-61	0.520	1.30	1.25~1.35
Rheumatoid arthritis (RA)	L04: Immunosuppressants	0~5.00E-08	272602	0.015	0.011	3.2E-93	0.586	3.18	2.74~3.68
		0~1.00E-05			0.012	2.3E-100	0.589	3.23	2.79~3.75
		0~1.00E-04			0.012	1.1E-102	0.590	3.47	2.99~4.04
		0~1.00E-03			0.013	1.3E-108	0.593	3.34	2.88~3.88
		0~1.00E-02			0.012	2.2E-104	0.593	3.28	2.83~3.80
		0~5.00E-02			0.010	2.9E-83	0.583	2.90	2.51~3.35
		0~1.00E-01			0.008	3.3E-67	0.573	2.52	2.19~2.91
		0~5.00E-01			0.006	4.9E-52	0.563	2.28	1.99~2.63
Femoral neck bone mineral density (BMD) [d]	M05B: Drugs affecting bone structure and mineralization	0~5.00E-08	215668	0.036	0.005	6.5E-70	0.558	2.09	1.88~2.32
		0~1.00E-05			0.007	2.5E-93	0.568	2.26	2.04~2.51
		0~1.00E-04			0.005	1.3E-62	0.555	1.88	1.69~2.08
		0~1.00E-03			0.004	2.7E-52	0.548	1.93	1.74~2.14
		0~1.00E-02			0.004	1.1E-48	0.548	1.74	1.57~1.93
		0~5.00E-02			0.004	4.7E-49	0.547	1.85	1.67~2.05
		0~1.00E-01			0.004	1.9E-48	0.546	1.83	1.66~2.03
		0~5.00E-01			0.004	4.6E-47	0.546	1.76	1.59~1.95
Migraine	N02C: Antimigraine preparations	0~5.00E-08	119844	0.046	0.012	5.1E-102	0.584	2.61	2.30~2.96
		0~1.00E-05			0.013	9.1E-110	0.585	3.08	2.70~3.51
Major depression (MD)	N06A: Antidepressants	0~5.00E-08	304162	0.111	0.001	7.6E-24	0.517	1.18	1.13~1.25
		0~1.00E-05			0.002	5.7E-54	0.525	1.39	1.32~1.47
		0~1.00E-04			0.002	5.0E-62	0.528	1.36	1.29~1.43
		0~1.00E-03			0.003	6.9E-101	0.535	1.55	1.48~1.64
		0~1.00E-02			0.004	4.9E-120	0.539	1.55	1.48~1.64
		0~5.00E-02			0.004	5.7E-134	0.540	1.70	1.61~1.79
		0~1.00E-01			0.004	1.6E-128	0.540	1.67	1.59~1.76
		0~5.00E-01			0.004	2.5E-134	0.540	1.67	1.59~1.76
Asthma	R03A: Adrenergics inhalants	0~5.00E-08	176445	0.164	0.013	1.6E-300	0.567	2.27	2.15~2.41
		0~1.00E-05			0.013	1.6E-301	0.567	2.29	2.16~2.43
		0~1.00E-04			0.013	1.8E-305	0.568	2.26	2.13~2.39
		0~1.00E-03			0.012	2.0E-277	0.565	2.25	2.12~2.38
		0~1.00E-02			0.011	4.1E-251	0.560	2.19	2.07~2.32
		0~5.00E-02			0.010	7.6E-228	0.558	2.08	1.96~2.20
		0~1.00E-01			0.009	3.2E-214	0.557	1.93	1.82~2.04
		0~5.00E-01			0.009	1.3E-201	0.555	2.00	1.89~2.12
Asthma	R03BA: Glucocorticoid	0~5.00E-08	205700	0.084	0.009	1.7E-172	0.562	2.21	2.05~2.37
		0~1.00E-05			0.008	3.5E-162	0.561	2.11	1.97~2.27
		0~1.00E-04			0.008	8.5E-167	0.562	2.00	1.86~2.14
		0~1.00E-03			0.007	1.9E-145	0.557	1.96	1.82~2.10
		0~1.00E-02			0.006	3.0E-122	0.551	2.01	1.87~2.16
		0~5.00E-02			0.006	2.7E-111	0.549	1.87	1.74~2.01
		0~1.00E-01			0.005	2.0E-102	0.548	1.77	1.65~1.90
		0~5.00E-01			0.005	1.5E-91	0.546	1.74	1.62~1.86

[a] For each discovery trait, the data are used from **Supplementary Table 3**.
[b] The odds ratio of taking medications for participants with genetic risk score at 10th decile compared with participants with genetic risk score at 1st decile.
[c] Confidence interval for the odds ratio at 10th decile.
[d] Effect size for each SNP from published GWAS summary statistics are changed into reverse direction.

Supplementary Table 5. The SNP-based heritability (h^2) of the 23 medication-taking traits, representing the proportion of variance of the case/control individuals in the UK Biobank attributable to genome-wide SNPs.

Medication category	h^2	h^2 SE	h^2 liability [a]	h^2 SE liability [a]	Lambda GC	Mean Chi^2	Intercept	Intercept SE	Ratio	Ratio SE	No. of case	No. of control
A02B	0.09	0.005	0.149	0.008	1.25	1.26	1.02	0.008	0.09	0.029	53137	79230
A10	0.05	0.003	0.203	0.014	1.25	1.32	1.04	0.010	0.12	0.030	15272	290641
B01A	0.05	0.004	0.084	0.007	1.15	1.18	1.02	0.007	0.10	0.042	67653	85986
C01D	0.01	0.002	0.115	0.018	1.10	1.09	1.02	0.007	0.24	0.077	5546	237113
C02	0.02	0.004	0.109	0.019	1.10	1.08	1.02	0.008	0.25	0.090	6431	145949
C03	0.10	0.006	0.231	0.014	1.37	1.53	1.05	0.012	0.10	0.022	34453	194633
C07	0.07	0.004	0.160	0.011	1.25	1.33	1.04	0.010	0.11	0.029	31700	192324
C08	0.11	0.006	0.242	0.013	1.37	1.49	1.05	0.010	0.10	0.021	31904	172474
C09	0.13	0.006	0.238	0.011	1.49	1.72	1.09	0.012	0.12	0.017	62752	174778
C10AA	0.07	0.006	0.136	0.010	1.31	1.46	1.05	0.011	0.10	0.023	73475	216910
H03A	0.07	0.006	0.218	0.020	1.25	1.49	1.06	0.013	0.13	0.027	24832	280750
L04	0.01	0.002	0.079	0.025	1.05	1.04	1.01	0.006	0.18	0.162	3954	268648
M01A	0.06	0.004	0.093	0.007	1.20	1.21	1.02	0.008	0.09	0.036	74150	90370
M05B	0.02	0.003	0.110	0.014	1.10	1.10	1.01	0.008	0.09	0.076	7870	207798
N02A	0.15	0.008	0.256	0.015	1.20	1.25	1.02	0.008	0.07	0.031	22982	55826
N02BA	0.06	0.005	0.088	0.009	1.10	1.14	1.01	0.007	0.09	0.054	61583	50427
N02BE	0.06	0.004	0.098	0.006	1.20	1.25	1.02	0.008	0.09	0.032	83218	96592
N02C	0.05	0.005	0.202	0.024	1.10	1.11	1.00	0.007	< 0	-	5521	114323
N06A	0.03	0.002	0.072	0.006	1.15	1.17	1.01	0.007	0.07	0.044	33757	270405
R03A	0.08	0.006	0.179	0.014	1.20	1.31	1.03	0.009	0.10	0.030	28880	147565
R03BA	0.03	0.004	0.114	0.012	1.15	1.17	1.03	0.008	0.17	0.048	17352	188348
R06A	0.03	0.004	0.100	0.011	1.10	1.11	1.01	0.007	0.05	0.064	13984	137652
S01E	0.05	0.006	0.235	0.028	1.10	1.12	1.01	0.007	0.06	0.062	5215	95653

[a] The h^2 on the sample scale has phenotypic variance approximately $P * (1 - P)$, where P is the proportion of the sample that takes the medication. We note that h^2 increases with P. Transformation to the liability scale assumes that the population risk of taking the medication, K is the same as the sample risk P, $K = P$. The transformation to the liability scale assumes an underlying normally distributed phenotype of liability to medication-taking.

Supplementary Table 6. Identified medication category associated-genes encoding therapeutic-effect targets for treating indications relevant to medications from corresponding medication category [a].

Gene	Medication categories	Protein name	Drug (the first 4 ATC level)	Mechanism of action
NDUFS3	A10	NADH dehydrogenase [ubiquinone] iron-sulfur protein 3, mitochondrial	Metformin (A10BA)	Mitochondrial complex I (NADH dehydrogenase) inhibitor
KCNJ11	A10	ATP-sensitive inward rectifier potassium channel 11	Acetohexamide |Chlorpropamide |Glimepiride |Glipizide |Glyburide |Tolazamide |Tolbutamide |Nateglinide |Repaglinide (A10BB |A10BX)	Sulfonylurea receptor 1, Kir6.2 blocker
PPARG	A10	Peroxisome proliferator-activated receptor gamma	Pioglitazone |Rosiglitazone |Troglitazone (A10BG)	Peroxisome proliferator-activated receptor gamma agonist
KCNH2	C07	Potassium voltage-gated channel subfamily H member 2	Sotalol (C07AA)	HERG blocker
CACNA1D	C08	Voltage-dependent L-type calcium channel subunit alpha-1D	Amlodipine |Clevidipine |Felodipine |Isradipine |Nicardipine |Nifedipine |Nimodipine |Nisoldipine |Verapamil |Diltiazem |Bepridil (C08CA |C08DA |C08DB |C08EA)	Voltage-gated L-type calcium channel blocker, Voltage-gated calcium channel blocker
CACNB2	C08	Voltage-dependent L-type calcium channel subunit beta-2	Bepridil (C08EA)	Voltage-gated calcium channel blocker
ACE	C09	Angiotensin-converting enzyme	Benazepril |Captopril |Enalapril |Fosinopril |Lisinopril |Moexipril |Perindopril |Quinapril |Ramipril |Spirapril |Trandolapril (C09AA)	Angiotensin-converting enzyme inhibitor
HMGCR	C10AA	3-hydroxy-3-methylglutaryl-coenzyme A reductase	Atorvastatin |Cerivastatin |Fluvastatin |Lovastatin |Pitavastatin |Pravastatin |Rosuvastatin |Simvastatin (C10AA)	HMG-CoA reductase inhibitor
TNF	L04	Tumor necrosis factor	Adalimumab |Certolizumab pegol |Etanercept |Golimumab |Infliximab (L04AB)	TNF-alpha inhibitor
GUCY1B3	C08	Guanylate cyclase soluble subunit beta-1	Nitroprusside |Riociguat (C02DD |C02KX)	Soluble guanylate cyclase activator, Soluble guanylate cyclase positive allosteric modulator
GUCY1A3	C08	Guanylate cyclase soluble subunit alpha-3	Riociguat (C02KX)	Soluble guanylate cyclase positive allosteric modulator
PCSK9	C10AA	Proprotein convertase subtilisin/kexin type 9	Alirocumab |Evolocumab (C10AX)	Subtilisin/kexin type 9 inhibitor
NPC1L1	C10AA	Niemann-Pick C1-like protein 1	Ezetimibe (C10AX)	Niemann-Pick C1-like protein 1 inhibitor
ABCA1	C10AA	ATP-binding cassette sub-family A member 1	Probucol (C10AX)	ATP-binding cassette sub-family A member 1 inhibitor
TRPM8	N02BE, N02C	Transient receptor potential cation channel subfamily M member 8	Menthol	Transient receptor potential cation channel subfamily A member 1 and subfamily M member 8 opener
IL5	R03A	Interleukin-5	Reslizumab |Mepolizumab (R03DX)	Interleukin-5 inhibitor

[a] Contents above bold black line show the therapeutic effect target genes for medications classified in the medication category while contents below bold black line show the therapeutic effect target genes for treating indications relevant to medications from corresponding medication category.

Supplementary Table 7. Therapeutic effect target genes for drugs ever or currently in clinical trial for the treatment of diseases corresponding to each medication category.

Gene	Medication categories	Protein name	Drug	Mechanism of action	Indication	Phase of clinical trial	Clinical trial identifier
CYP11B2	C03; C08; C09	Cytochrome P450 11B2	Osilodrostat	Aldosterone synthase inhibitor	Essential Hypertension	2	NCT00758524
EDNRA	C09	Endothelin receptor ET-A	Darusentan	Endothelin receptor ET-A antagonist	Hypertension	3	NCT00330369
CETP	B01A; C10AA	Cholesteryl ester transfer protein	Anacetrapib	Cholesteryl ester transfer protein inhibitor	Hypercholesterolemia	3	NCT01860729
IL4	R03A	Interleukin-4	Pascolizumab	Interleukin-4 inhibitor	Asthma	2	NCT00024544
IL4R	R03A; R03BA	Interleukin-4 receptor subunit alpha	Dupilumab	Interleukin-4 receptor subunit alpha antagonist	Asthma	3	NCT02414854
IL13	R03A; R03BA	Interleukin-13	Lebrikizumab	Interleukin-13 inhibitor	Asthma	3	NCT02104674
TSLP	R03A; R03BA; R06A	Thymic stromal lymphopoietin	Tezepelumab	Thymic stromal lymphopoietin inhibitor	Asthma	3	NCT03347279
TNF	R03A; R03BA	Tumor necrosis factor	Etanercept	TNF-alpha inhibitor	Asthma	2	NCT00141791

Supplementary Table 8. Connectivity score between the signature of perturbagen and knock-down gene.

A10 [a]		Cell line						
		PC3	VCAP	HCC515	HCC515	HT29	VCAP	HT29
		Medication name [b]						
		Acarbose	Glimepiride	Glipizide	Metformin	Nateglinide	Repaglinide	Tolbutamide
**Knock-down gene**	*IDE*	95.63 [c]	91.23	95.18	92.69	90.6	96.27	94.76

C09 [a]		Cell line							
		HEPG2	MCF7	PC3	HEPG2	A549	HA1E	HCC515	PC3
		Medication name							
		Candesartan	Enalapril	Enalapril	Quinapril	Ramipril	Telmisartan	Telmisartan	Valsartan
**Knock-down gene**	*AGT*	96.69	93.69	98.14	97.57	93.37	95.77	91.36	93.37

[a] Code in the cell represents medication category.
[b] Text in the next line represent medications from the medication category.
[c] Connectivity scores between the signature of perturbagen and knock-down gene in different cell lines are derived from Touchstone tool (https://clue.io/touchstone) on Feb 24, 2019. As recommended, scores > 90 are strong scores to be considered as hypotheses for further study.

IDE (Entrez ID: 3416) was associated with taking A10 medications (diabetes drugs) in our analyses and the inhibitor of insulin-degrading enzyme (encoded by *IDE*) has shown anti-diabetic activity (Majanti *et al*, 2014). *AGT* (Entrez ID: 183) was associated with taking C07 (beta-blockers) and C09 (renin-angiotensin agents) and a liver-selective angiotensinogen inhibitor from a recent study shows improvements in efficacy and tolerability on hypertension (Mullick *et al*, 2017). Since inhibition of insulin-degrading enzyme (encoded by *IDE*) and angiotensinogen (encoded by *AGT*) showed medication category relevant therapeutic effects, we also used the CLUE Touchstone tool (https://clue.io/touchstone) (Subramanian *et al*, 2017) to check the correlation between signatures of medication-taking from their corresponding medication category and knocking down the *IDE* and *AGT* gene in different cell lines. *IDE* and *AGT* showed a high correlation (using the criterion of Connectivity score > 90 as recommended by CLUE Connectopedia) with corresponding medication categories (above), suggesting that these genes may be involved in how these medications exert effect or in the etiology of medication-related diseases.

Maianti JP, McFedries A, Foda ZH, et al. Anti-diabetic activity of insulin-degrading enzyme inhibitors mediated by multiple hormones. Nature 2014;511:94.
Mullick AE, Yeh ST, Graham MJ, Engelhardt JA, Prakash TP, Crooke RM. Blood Pressure Lowering and Safety Improvements With Liver Angiotensinogen Inhibition in Models of Hypertension and Kidney Injury. Hypertension 2017;70:566.
Subramanian A, Narayan R, Corsello SM, et al. A Next Generation Connectivity Map: L1000 Platform and the First 1,000,000 Profiles. Cell 2017;171:1437-52.e17.

Supplementary Table 9. Number of individuals diagnosed with drug induced adverse side effect from UKB ICD10 classification (Data field: 41202 and 41204).

ICD10 Coding [a]	Meaning [a]	Number of Main [b]	Number of Secondary [c]
D521	Drug-induced folate deficiency anaemia	0	0
D590	Drug-induced autoimmune haemolytic anaemia	6	4
D592	Drug-induced nonautoimmune haemolytic anaemia	3	1
D611	Drug-induced aplastic anaemia	7	23
E064	Drug-induced thyroiditis	1	1
E160	Drug-induced hypoglycaemia without coma	32	28
E231	Drug-induced hypopituitarism	1	2
E242	Drug-induced Cushing's syndrome	4	27
E273	Drug-induced adrenocortical insufficiency	15	24
E661	Drug-induced obesity	0	2
G211	Other drug-induced secondary parkinsonism	3	12
G240	Drug-induced dystonia	6	5
G251	Drug-induced tremor	6	13
G254	Drug-induced chorea	0	0
G256	Drug-induced tics and other tics of organic origin	0	0
G444	Drug-induced headache, not elsewhere classified	31	23
G620	Drug-induced polyneuropathy	5	70
G720	Drug-induced myopathy	8	26
H263	Drug-induced cataract	12	2
J702	Acute drug-induced interstitial lung disorders	1	0
J703	Chronic drug-induced interstitial lung disorders	2	0
J704	Drug-induced interstitial lung disorder, unspecified	5	7
K853	Drug-induced acute pancreatitis	7	2
L105	Drug-induced pemphigus	0	0
L640	Drug-induced androgenic alopecia	0	2
M1020-M1029	Drug-induced gout	1	3
M320	Drug-induced systemic lupus erythematosus	1	10
M8040-M8049	Drug-induced osteoporosis with pathological fracture	5	5
M8140-M8149	Drug-induced osteoporosis	15	199
M8350-M8359	Other drug-induced osteomalacia in adults	0	0
R502	Drug-induced fever	16	8

[a] Both the coding and the meaning were derived from Data-coding 19 of UKB.
[b] Number of individuals were counted using ICD10 main diagnoses data (Data field: 41202) of UKB.
[c] Number of individuals were counted using ICD10 secondary diagnoses data (Data field: 41204) of UKB.

Supplementary References

1 Bulik-Sullivan, B. K. *et al.* LD Score regression distinguishes confounding from polygenicity in genome-wide association studies. *Nature genetics* **47**, 291-295, (2015).
