# Supplementary material for: Genome-wide association study of medication-use and associated disease in the UK Biobank
Source: Nat Commun. 2019 Apr 23;10:1891. doi: 10.1038/s41467-019-09572-5 (PMC6478889; doi:10.1038/s41467-019-09572-5)
Supplement: Supplementary file 2 — Description of Additional Supplementary Files [file 41467_2019_9572_MOESM2_ESM.pdf]

Description of Additional Supplementary Files

- Supplementary Data 1. Active ingredients and ATC code of medications in UK Biobank.
- Supplementary Data 2. The independent SNPs ($P \leq$ 5E-8) associated with the 23 medication-taking traits.
- Supplementary Data 3. Results of partitioning the 23 traits' SNP-heritability to 205 tissues/cell types using cell type specific analyses.
- Supplementary Data 4. Results of SMR analysis for the 19 medication-taking traits.
- Supplementary Data 5. Results of gene-based association analyses.
- Supplementary Data 6. Results of gene sets enrichment analysis at FDR < 5%.
- Supplementary Data 7. Genetic correlation of the 23 medication traits and 21 related diseases/traits.
- Supplementary Data 8. Mendelian Randomization analysis results using the GSMR method and SNPs associated with 15 diseases/traits as instrument (trait1) to investigate the hypothesis of causality of the 23 medication-taking traits (trait 2).

1.
